# Funds Reimbursement of High-Cost Drugs in Gastrointestinal Oncology: An Italian Real Practice 1 Year Experience at the National Cancer Institute of Naples

**DOI:** 10.3389/fpubh.2018.00291

**Published:** 2018-10-12

**Authors:** Monica Capozzi, Chiara De Divitiis, Alessandro Ottaiano, Tramontano Teresa, Maurizio Capuozzo, Piera Maiolino, Gerardo Botti, Salvatore Tafuto, Antonio Avallone, The Abdominal Oncology Group, Rossana Casaretti

**Affiliations:** ^1^Department of Abdominal Oncology, National Cancer Institute, Istituto Nazionale Tumori di Napoli, Fondazione “G. Pascale”, Naples, Italy; ^2^Pharmacy Unit, National Cancer Institute, Istituto Nazionale Tumori di Napoli, IRCCS Fondazione “G. Pascale”, Naples, Italy; ^3^Pharmacy Unit, ASL Naples 3 South, Herculaneum, Naples, Italy; ^4^Pathology Unit, National Cancer Institute, Istituto Nazionale Tumori di Napoli, IRCCS Fondazione “G. Pascale”, Naples, Italy

**Keywords:** targeted drugs, reimbursement, public health, managed entry agreements, gastro-intestinal cancers, multidisciplinary team

## Abstract

**Introduction:** The therapeutic scenario of Oncology is enriching of innovative agents which are determining an increase in public expenditure because of their high cost. In Italy, a web-based government Registry is used to monitor the clinical use of these drugs and, in later phases, to obtain funds reimbursement according to specific economic agreements with companies.

**Methods:** A health policy expert Pharmacist was included in the multidisciplinary team of the Department of Abdominal Oncology of the National Cancer Institute (NCI) of Naples “G. Pascale Foundation” in order to improve the management of the Registry for oncologic drugs monitoring. Pharmacist activities were: basal data registration, prescription appropriateness, drug request, response monitoring, toxicity reporting, follow-up, reimbursement request. These activities were conducted in strict interrelation with clinicians. The source of data were medical records and a web-based national reimbursement platform. The analysis of the economic impact of this strategy was descriptive and it was indicated as resources recovery comparing 2 years: 2015 vs. 2016. The currency reference used was the Euro (€).

**Results:** A total of 932 patients were followed-up and registered, 365 treatments are ongoing at the Department of Abdominal Oncology (NCI of Naples, Italy). The most prescribed biologic drug in advanced gastrointestinal cancers was bevacizumab. Compared to the year 2015, in 2016 we recorded a strong increase of reimbursements: EUR 881.712,42 vs. EUR 214.554,98.

**Conclusions:** We suggest that the reimbursement process can be improved when a health policy reimbursement professional Pharmacist is integrated in the multidisciplinary team along with clinicians.

## Introduction

The therapeutic scenario of Oncology is enriching of innovative agents which are determining an increase in public expenditure because of their high cost (1). Biologic drugs in oncology are the fastest-growing pharmacological category worldwide; the increase in their use is paralleled by the progressive increase in the comprehension of the cancer biology. In the future, biologic target-based drugs will substitute “conventional” chemotherapies because of higher specificity against cancer cells (more on target interactions) and lower toxicity profile (less off target interactions). To date, in gastrointestinal oncology, some monoclonal antibodies (cetuximab, panitumumab, bevacizumab, ramucirumab, trastuzumab), a recombinant fusion protein (aflibercept) and a small molecule (regorafenib) are available to treat patients with metastatic disease. However, new biologics continue to emerge particularly in the field of cancer immunology. The steep prices of biologics concern researchers, clinicians, patients, and it is due to many factors (i.e. patents, intellectual property, competition, production costs, etc.) whose description and discussion are beyond the scope of the present report. In last years, many efforts have been pursued by the Healthcare Systems of the European countries to optimize the national pharmaceutical expenditure ensuring the access to these innovative and highly expensive treatments to patients. The National Health Authorities are called upon to determine the price of the new drugs in relation to their effectiveness and innovativeness; however, the data at the time of authorization of medicinal products for marketing by European Medicines Agency (EMA) are often insufficient to accurately estimate the effects (efficacy and toxicity) in real practice (2). Managed Entry Agreements (MEA) are specific economic instruments in order to facilitate the access to these high-cost innovative agents (“access policy”) (3–9). MEA can be divided into two main groups: financial-based and performance-based. The first consists on price-volume discount (manufacturers pay-back the excess of public established threshold expenditure) and dose-capping scheme (manufacturers refund the overdoses of established doses required per year per drug). The second, “performance-based” consists on outcome-based evaluations: the efficacy data are collected and the cost is reduced or reimbursed according to the outcome obtained in real practice. However, details about MEA (entity of reimbursements, timings, outcomes, etc.) cannot be revealed because of their private nature.

In Italy, the *Agenzia Italiana del Farmaco* (AIFA, Italian governative agency for pharmaceutical products) negotiated different agreements with pharmaceutical industry. The contractual arrangements include both financial- and performance-based agreements (10). The new authorized drugs are immediately included in a specific “registry” of AIFA: a government web-based tool in order to monitor appropriateness, use, toxicity and efficacy of pharmaceuticals. One hundred thirty innovative drugs are currently monitored on Italian Registry.

In this report, we show a real practice experience of reimbursement at the Department of Abdominal Oncology of the National Cancer Institute of Naples.

## Methods

An expert Pharmacist (MC) was involved in a project at the Department of Abdominal Oncology of the National Cancer Institute of Naples “ G. Pascale Foundation” in order to evaluate the economic impact of improving the management of AIFA Registry for high-cost innovative drugs (including monoclonal antibodies or small molecules inhibiting the proliferative signals or with antiangiogenic properties or differentiating agents or inhibitors of metastasis/invasiveness or drugs with multiple mechanisms of action). The selected drugs were: bevacizumab, cetuximab, panitumumab, trastuzumab. Reasons for excluding nab-paclitaxel, ramucirumab, aflibercept and regorafenib are described in results. During the entire 2016, management of AIFA registry was done in strict collaboration with a health policy reimbursement expert Pharmacist.

The Pharmacist was involved in following specific data entry activities: (i) patients' and disease characteristics registration (basal data), (ii) evaluation of the eligibility criteria (prescription appropriateness), (iii) registration of code, type, number and quantity of drug vials dispensed (drug request), (iv) disease status monitoring (response monitoring), (v) adverse reactions reporting (toxicity reporting), (vi) “end of treatment” module (follow-up). Finally, the Pharmacist managed the request forms for reimbursement (reimbursement request) interacting with companies and following that process from the submission to the approval of refunding. These activities were conducted in strict interrelation with clinicians.

The source of data of this report were medical records and the web-based national reimbursement platform. The analysis was descriptive and the outcome of this study was indicated as cumulative recovery of resources comparing 2 years: 2015 vs. 2016. The currency reference used was the Euro (€). Statistical inference was not applied given the high differences observed.

## Results

The Department of Abdominal Oncology of the National Cancer Institute of Naples is particularly devoted to the diagnosis and treatment of cancers of the colon, rectum, stomach and pancreas. The medical division is actively involved in data entry and updating of the AIFA Registry in accordance with national legislation (Decree Law 6 July 2012 n. 95, “Spending review Law”). Table 1 shows the most important innovative drugs used. For the medicinal product drugs Cetuximab and Panitumumab the monitoring activity has been stopped since October 2th, 2016 and February 27 th, 2017, respectively.

**Table 1:** Innovative drugs and their mechanism of action.

**Drug name**	**Molecular classification**	**Mechanism of action**	**Type of cancer**
Aflibercept	Recombinant fusion protein	Binds to VEGF-A/B, PlGF	Colorectal
Bevacizumab	Humanized mAb IgG1	Binds to VEGFs	Colorectal
Cetuximab	Chimeric mouse-human mAbIgG1	Binds to EGFR	Colorectal
Nab-Paclitaxel	10-Deacetylbaccatin-type molecule albumin-stabilized nanoparticle	Stabilization of the microtubule polymer	Pancreas
Panitumumab	Humanized mAb IgG2	Binds to EGFR	Colorectal
Ramucirumab	Humanized mAb IgG1	Binds to VEGF receptor 2	Gastric
Regorafenib	4-(4-(3-(4-chloro-3- (trifluoromethyl)phenyl)ureido)-3-fluorophenoxy)-N-methylpicolinamide.	Multi-kinase inhibitor (VEGFR1,2 and 3, TIE-2, PDGFR, c-kit, ret, raf-1)	Colorectal
Trastuzumab	Humanized mAb IgG1	Binds to HER2	Gastric

*mAb, monoclonal antibody; VEGF, Vascular Endothelial Growth Factor; PlGF, Placental Growth Factor; EGFR, Epithelial Growth Factor Receptor; TIE-2, Tyrosine kinase with immunoglobulin-like and EGF-like domains 2; PDGFR, Platelet Derived Growth Factor Receptor; HER, Human Egf Receptor*.

Actually, a total of 932 patients are followed-up and 365 treatments are ongoing for gastrointestinal oncology specialties MEA-repayable. The overall picture of the cumulative number of treatments from January 2013 to December 2016 is shown in Table 2. The most prescribed biologic drug in advanced disease was bevacizumab. There was an increase in the use of panitumumab and of new authorized drugs (nab-paclitaxel and ramucirumab). By contrast, a significant decrease was registered for cetuximab. However, at the date of presentation of this report, MEA were not yet activated for Nab-paclitaxel and Ramucirumab so that these drugs have been excluded from the comparative analysis (2015 vs. 2016). Furthermore, also aflibercept was excluded because MEA reimbursement started in the late 2015. Data cannot be extracted for regorafenib because the web-based registration and monitoring started in January 2017.

**Table 2:** Enrolment of patients in the AIFA Registry according to specific drugs and years (from 2013 to 2016).

**Drug**	**Managed Entry Agreements (MEA)**	**Patients treated at 2015^a^**	**Patients treated at 2016**	**Absolute treatments increase in 2016**
Bevacizumab	Financial-based	373	473	100
Cetuximab	Performance-based	169	175	6
Panitumumab	Performance-based	86	139	53
Trastuzumab	Performance-based	36	45	9

a *From January 2013*.

**Figure 1:**
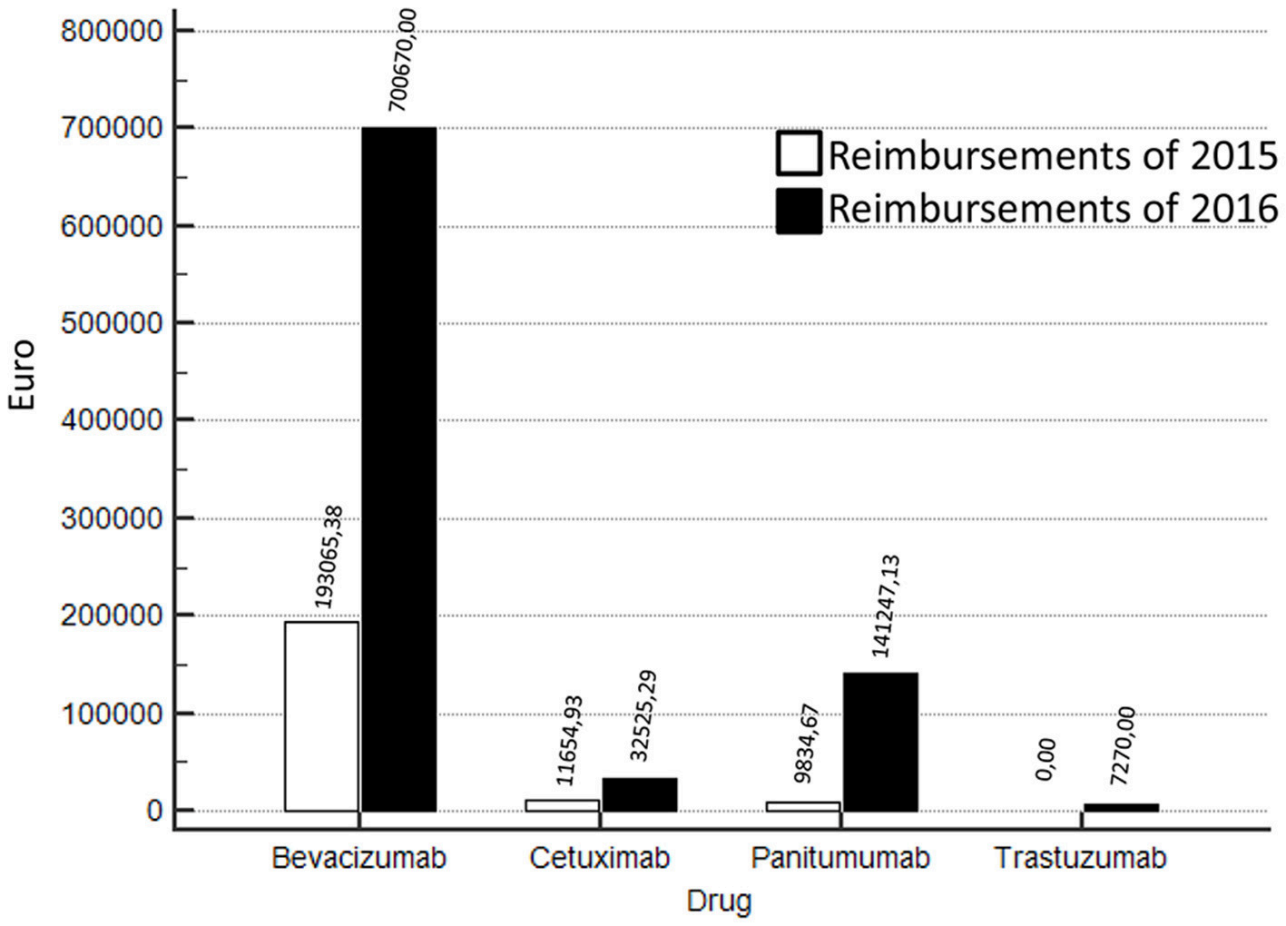
Reimbursements comparisons 2016 vs 2015 according to different biologics (bevacizumab, cetuximab, panitumumab, trastuzumab).

Compared to the year 2015, in 2016 a strong increase of funds reimbursement was observed. In twelve months of project activity (from March 2016 to February 2017) EUR 881.712,42 have been reimbursed against the EUR 214.554,98 of 2015 (Figure 1). In particular, in 2016, 54 Bevacizumab, 26 Cetuximab, 10 Trastuzumab, 18 Panitumumab treatments were closed and successfully submitted for reimbursement. In Figure 1 we show also the comparison between 2015 vs. 2016 in terms of reimbursements for trastuzumab (advanced HER2+ gastric cancer), panitumumab, cetuximab (advanced RAS wild type colorectal cancer), and bevacizumab (advanced colorectal cancer). The increase in reimbursement was not attributable to increase of treated patients in 2016 compared to previous years (Table 2). In fact, the use of cetuximab and trastuzumab was reduced in 2016; the reduction of cetuximab was paralleled by a significant increase of panitumumab.

Most of reimbursements was attributable to bevacizumab which was stable over the observed years. Additionally, in 2015, the procedures of reimbursement were already active. Thus, the differences in results are attributable to a more careful monitoring activity and more timely submission of requests for reimbursement to pharmaceutical companies.

Our study suggest that the AIFA registry is a real chance of founding if managed by qualified professionals.

## Discussion

The Healthcare System has to satisfy the challenge of optimizing the national public pharmaceutical expenditure ensuring to patients the access to innovative treatments. In Italy, the AIFA Registry assess the patient's eligibility for treatment, collects epidemiological data, drug safety and efficacy profile. This should ensure the appropriate use of medicines as recommend by guidelines and provide data about the “real world” efficacy of the drugs. The AIFA Registry was established in 2005 and completely renewed in 2013 and it belongs to the Information System of the National Health Service that estimates, through the data collected, the benefit/risk and cost/effectiveness ratios of pharmaceuticals. The AIFA Registry is also part of a broader European program of Health Technology Assessment (HTA) representing the multidisciplinary approach to analyze the effects of therapeutic innovation in clinical practice in order to reduce public expenditure (11). However, the application of MEA requires the correct use of monitoring, in accordance with very specific requirements and deadlines regarding the restaging of the disease, the number of therapy cycles, the monitoring and reporting of therapy response, the timely communication of adverse events, and correct follow-up information.

In the present study, a Pharmacist was involved in the multidisciplinary team of the Abdominal Oncology Department of the National Cancer Institute in order to implement specific activities related to the AIFA registry for the patients with advanced gastrointestinal cancer going to start innovative therapy. In particular the Pharmacist was committed to entry, manage and discuss with clinicians the basal data, prescription appropriateness, drug requests, response monitoring, toxicity reporting, “end of treatment” module. Finally, the Pharmacist managed all the process of reimbursement request from the submission to the approval of refunding. Interestingly, after including this professional figure in the multidisciplinary team, from March 2016 to February 2017, there was an increase in reimbursements at the Department of Abdominal Oncology of the National Cancer Institute of Naples; in fact, EUR 881.712,42 were reimbursed against the EUR 214.554,98 of 2015 (Figure 1) and that that increase was not attributable to the number of patients (Table 2).This was related to improvements of all activities related to AIFA registry management and reimbursement requests. Optimization of costs along with clinical efficacy is a goal of sanitary systems and many experiences in literature have already indicated strategies both in oncology and other therapeutic areas in order to achieve this objective: patients' selection (12–14), drug-days (15–17), monitoring of prescription appropriateness (18–20) and MEA (10).

Uncertainty of clinical outcomes and high costs are among the main challenges of innovative drugs; the first issue needs to be faced with intensive clinical and translational research, the second one has prompted the adoption of refunding systems including MEA. The results of this report indicate that MEA are an important source of reimbursement for innovative drugs but this system requires highly skilled and dedicated personnel. In the new era of high-cost innovative biologic drugs, professionals figures, beside clinicians, should be involved in the management of economic-related issues of anti-neoplastic agents.

## Conclusion

In the present study we show that the reimbursement process of biologics in gastrointestinal oncology can be improved when a health policy reimbursement professional Pharmacist is integrated in the multidisciplinary team along with clinicians. The present study can be considered as an exploratory report lacking in literature data on the integration of Pharmacists into the clinical multidisciplinary teams. Improving the management of MEA-related issues could represent a successful strategy in the “real world” to improve reimbursements and finally reduce the costs of biologic drugs.

## Author contributions

CD, AO, GB, ST, and AA for enlistment and treatment of patients and prescription of drugs. MoC, TT, MaC, and PM for drug delivery and processing of pharmaceutical spending data. Abdominal Oncology Group were involved in treatment of patients and writing the manuscript.

### Conflict of interest statement

The authors declare that the research was conducted in the absence of any commercial or financial relationships that could be construed as a potential conflict of interest.
